# Superior Sensibility after Full Breast Reconstruction with Autologous Fat Transfer

**DOI:** 10.1097/PRS.0000000000010619

**Published:** 2023-04-28

**Authors:** Jamilla L. M. Wederfoort, Sander Schop, Lotte C. A. van der Broeck, Juliette E. Hommes, Sander M. J. van Kuijk, Floyd Timmermans, Jan Maerten Smit, Esther M. Heuts, Thijs de Wit, René R. W. J. van der Hulst, Andrzej A. Piatkowski

**Affiliations:** Maastricht, Amsterdam, Breda, and Venlo, the Netherlands; From the Departments of 1 Plastic, Reconstructive, and Hand Surgery; 4 Clinical Epidemiology and Medical Technology Assessment; 6 Surgery, Maastricht University Medical Center+; 2 NUTRIM School of Nutrition and Translational Research in Metabolism; 3 Faculty of Health, Medicine, and Life Sciences, Maastricht University; 5 Plastic, Reconstructive, and Hand Surgery, VU University Medical Centre Amsterdam; 7 Plastic, Reconstructive, and Hand Surgery, Amphia Hospital; 8 Plastic, Reconstructive, and Hand Surgery, VieCuri Medical Center.

## Abstract

**Background:**

With developments in screening and treatment, survival rates of breast cancer patients are increasing, and so is the number of women opting for breast reconstruction to improve their quality of life. One factor that could play an important role in improving the quality of life is breast sensibility. Therefore, the aim of this study was to explore breast sensibility in participants of the Breast Reconstruction with External Preexpansion and Autologous Fat Transfer versus Standard Therapy trial: an ongoing randomized controlled trial comparing breast reconstruction with autologous fat transfer (AFT) versus implant-based reconstruction (IBR).

**Methods:**

This study was conducted on participants of the Breast Reconstruction with External Preexpansion and Autologous Fat Transfer versus Standard Therapy trial who were at least 12 months after final surgery. Semmes-Weinstein monofilaments were used to measure skin sensibility in breast cancer patients who underwent breast reconstruction with either AFT or IBR following their mastectomy.

**Results:**

A total of 46 patients were included in this study, resulting in 62 breast reconstructions (28 AFT breasts and 34 IBR breasts). Significantly higher mean monofilament values were found for skin sensibility after AFT (−0.7; *P* < 0.001), clinically correlating to “diminished protective function,” as opposed to the IBR group, with clinical values indicating “loss of protective function.”

**Conclusions:**

In this study, the authors found that breast cancer patients who underwent a mastectomy had a significantly better sensibility of the breast following AFT for total breast reconstruction as compared with IBR. Larger studies that include null measurements are required to further explore these noteworthy results of AFT.

**CLINICAL QUESTION/LEVEL OF EVIDENCE:**

Therapeutic, II.

Breast cancer is the most prevalent malignancy in women worldwide, accounting for 27.6% of malignant cases in women across the globe, during the year of 2020.^1^ As the survival rate increases because of the development of advanced screening programs and early-onset treatment, many live with the aftermath of breast cancer treatment. This is often accompanied by an effect on quality of life (QoL), especially after surgical intervention.^2–4^ The two major surgical treatments are breast-conserving therapy and skin- or nipple-sparing mastectomy. In the Netherlands, a mastectomy is performed in 33% to 40% of breast cancer patients, of whom 20% undergo breast reconstruction.^5^

The primary aim of breast reconstruction is improving QoL. Breast reconstruction has been shown to increase QoL in breast cancer survivors, considering sexuality, femininity, and intimacy.^6^ In addition, the presence of sensibility of the breast has been associated with improved QoL.^7^ However, after breast reconstruction, sensibility of the breast is deteriorated, and one study showed the sensibility is especially decreased after implant-based breast reconstruction (IBR).^6,8^ This loss of sensibility can be devastating and even dangerous, because women can sustain severe burns or damage to the skin.^9^ Therefore, in recent years, studies have been conducted on how to improve sensibility of a reconstructed breast, especially in free flap breast reconstruction.^10–13^

Although autologous fat transplantation (AFT) is a rising breast reconstruction technique, other breast reconstruction methods, such as IBR or the deep inferior epigastric perforator (DIEP) flap, are well established and considered the accepted standards. Nonetheless, AFT has already proven to be minimally invasive and effective.^14–18^

Essentially, AFT uses autologous adipose tissue liposuction from donor sites that will be filtrated to separate the fat from the unwanted fluids (eg, blood, infiltrated saline) to then reinject into the breast.^14,16,17,19^ Although multiple sessions of AFT are necessary to reach a desired breast volume, high patient and surgeon satisfaction rates have been reported. Besides effectiveness in terms of volume and aesthetics, AFT may also stimulate tissue regeneration after radiation therapy, decrease pain levels, and ensure oncologic safety, thus depicting a favorable procedure.^20,21^

Breast sensibility is a key predictor for QoL following breast reconstruction after mastectomy.^7,9^ With that said, AFT benefits from its autologous features and possibly has an effect on pain and sensibility, and thus, predicted enhanced sensibility after AFT reconstruction will add to the beneficial effects of AFT and thus will have a greater ability to improve QoL in breast cancer patients.

So far, no studies have been published investigating sensibility of the skin following AFT for total breast reconstruction. Therefore, the objective of this study was to investigate sensibility of the breast following AFT reconstruction in breast cancer patients who underwent a mastectomy, as compared with IBR. It is hypothesized that sensibility of the breast following AFT will be superior to sensibility in breast cancer patients who underwent IBR after mastectomy.

## PATIENTS AND METHODS

The Strengthening the Reporting of Observational Studies in Epidemiology (STROBE) checklist for observational studies was adhered to for composing this article. (**See Appendix, Supplemental Digital Content 1**, which shows the STROBE checklist, http://links.lww.com/PRS/G391.) Ethical approval was obtained from the local medical ethical committee of Academic Hospital Maastricht/Maastricht University (METC14-2059). This study was an amendment for some of the participants of the original Breast Reconstruction with External Preexpansion and Autologous Fat Transfer versus Standard Therapy (BREAST) trial; additional informed consent for sensibility measurement was obtained from all participants. The BREAST trial is a multicenter randomized controlled trial running from November of 2015 to October of 2025 in seven participating centers across the Netherlands.^22^ All participants were included in a 1:1 stratified randomization, comparing AFT (cases) with IBR (controls). Randomization was done with a computer-generated randomization schedule. The main outcome for the BREAST trial is the QoL at 12 months following breast reconstruction, measured with the BREAST-Q questionnaire. In addition, the efficacy and safety of these breast reconstruction methods is studied.

### Study Design

Participants of the BREAST trial who reached at least 12 months after reconstruction were considered eligible for this breast sensibility study.^22^ Sensory testing for this prospective multicenter sensibility study was performed from October of 2018 to May of 2021 in three participating centers. Semmes-Weinstein monofilaments (SWM) were used for measurement by four independent researchers (J.L.M.W., L.C.A.v.d.B., S.S., and F.T.), who were not informed of type of breast reconstruction before measurement. The primary outcome was sensibility, measured at 12 months after the last surgical intervention for breast reconstruction. Correlation between breast volume and sensibility was the secondary outcome.

### Participants

Patients who met the inclusion and exclusion criteria of the BREAST trial, were included in one of three of the seven participating BREAST trial centers, and were at their final follow-up moment were approached to participate. (**See Appendix, Supplemental Digital Content 2**, which shows inclusion and exclusion criteria for the BREAST trial, http://links.lww.com/PRS/G392.)

### Surgical Procedures

All patients (both IBR and AFT) underwent a modified radical mastectomy before their assigned breast reconstruction commenced. Before each operation, patients assigned to the AFT group underwent preexpansion with the BRAVA device for 4 weeks. The vacuum pump was set at a cycling program between 0 and 60 mmHg, and patients were requested to wear the device for 10 hours per day and 24 hours per week, 7 days per week during the last 48 hours preoperatively. During AFT surgery, the Coleman technique was applied for liposuction and the fat was collected and filtered using the Puregraft collection bag.^23^ Thereafter, the fat was grafted into different pectoral layers and into the deep and superficial dermis of the breast. Serial lipofilling was performed until the desired breast volume was achieved. One-day postoperative patients were asked to wear the BRAVA device for 2 weeks at a low continuous pressure (−20 mmHg) for 10 hours per day. After a minimum of 10 weeks, the next AFT operation was planned.

The control group underwent two-phase IBR. During the first operation, a tissue expander (TE) was placed subpectorally. The TE was filled subsequently with saline during the outpatient visits until the desired volume was achieved. An operation was then planned to replace the TE with a subpectoral definitive implant.

### Data Management

Patient characteristics, such as age, body mass index, laterality, and previous IBR, and clinical data including follow-up duration, number of reconstruction operations, total breast volume, history of chemotherapy and/or lymphadenectomy, and complications were extracted from the electronic patient file.

### Sensory Testing

**Fig. 1.**
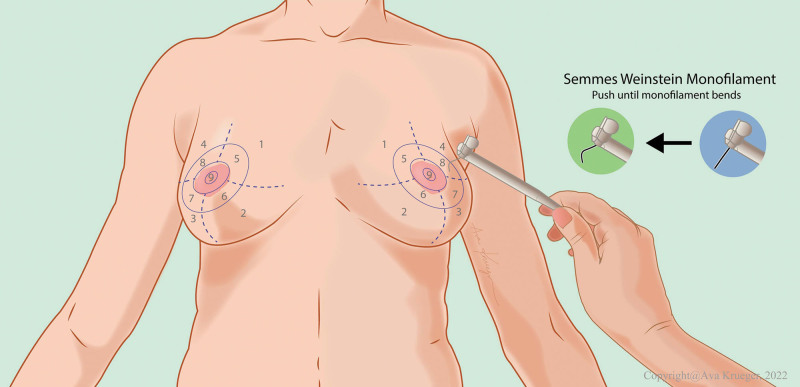
Predefined measurement areas of the breast and example of SWM testing. (Copyright © 2022 Ava Krueger, scientific illustrator.)

Sensory testing was performed using the 20-piece SWM set.^24,25^ Each monofilament, ranging in thickness with an index value from 1.65 to 6.65, represents the logarithmic force on a base 10 in mg necessary to bend the monofilament. Quantification of sensibility was performed through notation of first recognition of sensibility by the smallest monofilament, establishing the sensibility threshold. Anatomical references were used to define nine areas of the breast, as shown in Figure 1. Measurements were performed in a quiet room with patients lying in the supine position with their eyes closed. Sensibility was examined accordingly by applying perpendicular pressure to the same spot until the monofilament bent each time for a duration of 1.5 seconds, three times in succession, with intervals of 1.5 seconds, until noted by the participant. To prevent the patient from being able to predict a site, areas were tested in a random sequence.

SWM index values can be translated to clinical means with green representing “normal touch” (1.65 to 2.83), blue representing “diminished light touch” (3.22 to 3.61), purple representing “diminished protective function” (3.84 to 4.31), and red representing “loss of protective function” (4.56 to 6.45) and “deep pressure sensation only” (6.65).^26^ If a mean SMW index value fell between two clinical means, the first detectable value was chosen.

### Breast Volumes

The VECTRA 3D^XT^ imaging system was used to create three-dimensional images of breast reconstructions. It was assumed that at 12 months after final reconstruction surgery, breast volumes were stabilized; therefore, all images were taken at 12 months postoperatively. Patients were asked to stand in front of the camera with their arms abducted at 45 degrees. These 12-month images were collected from all centers, and breast volumes were measured using the VECTRA 3D^XT^ software. Two blinded independent researchers (J.W. and L.C.A.v.d.B.) used the VECTRA 3D^XT^ sculptor software to manually select landmarks on the thorax (sternal notch, right/left nipple, right/left areola, right/left midclavicula, right/left inframammary fold, right/left lateral mammary fold) to calculate breast volumes.

### Statistical Analysis

Statistical analyses were performed using IBM SPSS version 25. Categorical variables are shown as frequencies and percentages of total. Continuous variables are presented as means and standard deviations. As this study may represent a nonrandom subset of the original randomized BREAST trial, we did also test for baseline differences between groups. To test for between-group differences, the Pearson chi-square test or Fisher exact test were used. Continuous variables were tested using the independent samples *t* test.

Monofilament notation was done using their index values, marked on each rod. The mean and its standard deviations were computed for both groups, per location (areas 1 through 8) and for an overall score. Overall sensibility score was computed by obtaining the mean of all areas per patient. As some patients were included with a bilateral reconstruction, linear mixed-effects models were used to correct SWM values for baseline characteristics and differences in baseline characteristics between the groups, and to account for clustered observations (ie, bilateral surgery). Adjusted differences in SWM values between treatment groups are shown as adjusted crude differences with their 95% confidence intervals. We computed correlation coefficients to estimate the association between breast volume and SWM values.

## RESULTS

### Participants

The total cohort of three participating centers provided 67 eligible BREAST trial patients, of which 46 participants (24 AFT participants and 22 IBR participants) were included and examined in this study between October of 2018 and May of 2021. Because of the presence of bilateral reconstructions, 28 AFT and 34 IBR breasts were studied, accounting for a sample of 62 breasts. A flowchart for participants is shown. (**See Appendix, Supplemental Digital Content 3**, which shows the study flowchart showing how study size was reached, http://links.lww.com/PRS/G393.)

### Descriptive Data

Baseline characteristics for both groups are presented in Table 1. On average, patients in the AFT group were older (*P* = 0.030), and more patients underwent unilateral reconstruction than in the IBR group (*P* = 0.012). AFT breasts were smaller, with a mean difference of −135.4 (*P* < 0.001) and more operations were necessary to complete breast reconstruction. Other patient characteristics did not differ substantially between the groups.

**Table 1.** Baseline Characteristics

	AFT (%)	IBR (%)	*P*
No.	24	22	
Mean age ± SD, yr	54.0 ± 7.0	48.0 ± 11.1	0.030^a^
Mean BMI ± SD, kg/m²	23.9 ± 2.2	22.9 ± 2.4	0.178
History of reconstruction (IBR)^b^	4.0 (16.7)	6.0 (27.3)	0.484
History of chemotherapy	7.0 (29.2)	0.0 (0.0)	0.010^a^
Mean time after final surgery ± SD, mo	24.1 ± 13.3	29.9 ± 16.2	0.191
Mean time after mastectomy± SD, mo	51.1 ± 13.4	43.6 ± 12.5	0.099
Mean no. of operations ± SD	4.5 ± 0.8	2.0 ± 0.5	<0.001^a^
Lymphadenectomy	3.0 (12.5)	0.0 (0.0)	0.235
Mean volume per breast ± SD, cc	310.6 ± 144.0 (*n* = 28)^c^	446.0 ± 111.4 (*n* = 34)^c^	<0.001^a^

BMI, body mass index.

a Statistically significant.

b This entails the number of patients who underwent IBR and needed additional surgery to remove/exchange the implant because of an implant-related complication.

c Numbers are different from number of patients, because volume analysis was performed per breast and some patients underwent a bilateral reconstruction.

### SWM Values

**Fig. 2.**
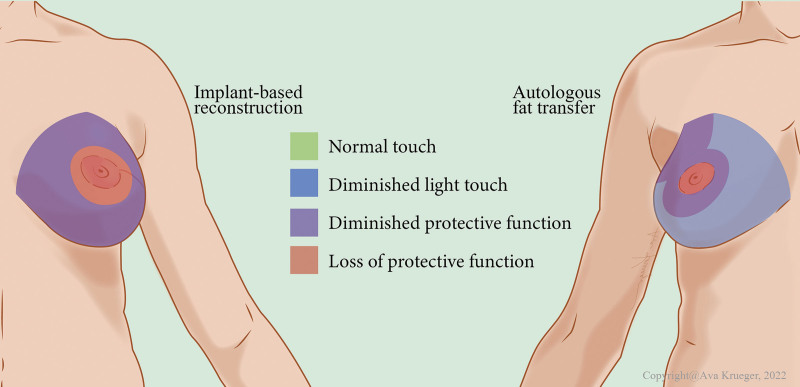
Interpretive illustration showing altered level of sensibility following AFT (*right*) and IBR (*left*). The index shows the definition of the colors, with *red* indicating loss of protective function. In this figure, we show the sensibility in the breast returns to diminished protective function following AFT reconstruction, whereas a larger area surrounding mamilla in IBR patients shows a loss of protective function. (Copyright © 2022 Ava Krueger, scientific illustrator.)

Mean index values of the monofilaments per area are shown in Table 2. With the exception of the upper medial and upper lateral regions of the breast, all areas had a significantly lower mean index value in the AFT group, indicating better sensibility. When considering all areas of the breast together, the mean value for the AFT group was also lower. The average threshold monofilament value of the AFT group was 3.84, associated with “diminished protective function,” whereas the mean threshold value for the IBR group was 4.56, associated with “loss of protective function.” Table 3 shows all crude coefficients and adjusted crude coefficient between the control group and AFT group. Figure 2 illustrates clinically defined levels of sensibility followed by AFT and IBR.

**Table 2.** Mean SWM Results per Area for the AFT and IBR Groups

	Anatomical Region	AFT	IBR	*P*
No.		28	34	
Area				
1	Upper medial	3.21 ± 0.79	3.66 ± 0.92	0.051
2	Lower medial	3.27 ± 0.73	3.82 ± 0.91	0.011^a^
3	Lower lateral	3.56 ± 0.79	4.16 ± 0.98	0.012
4	Upper lateral	3.64 ± 0.84	3.99 ± 1.03	0.161
5	Upper medial, center	3.97 ± 0.79	4.81 ± 0.67	<0.001^a^
6	Lower medial, center	4.00 ± 0.64	4.82 ± 0.87	<0.001^a^
7	Lower lateral, center	4.18 ± 0.75	5.04 ± 0.81	<0.001^a^
8	Upper lateral, center	4.09 ± 0.80	5.07 ± 0.79	<0.001^a^
9	Center mamilla	4.58 ± 0.76	5.50 ± 0.79	<0.001^a^
1–9	Total breast skin	3.84 ± 0.59	4.54 ± 0.63	<0.001^a^

a Statistically significant.

**Table 3.** Crude Coefficient and Adjusted Crude Coefficient between the Control Group and the AFT Group^a^

Area	Crude Coefficient	95% CI	*P*	Adjusted Crude Coefficient	95% CI	Adjusted *P*
1	−0.4	−0.9 to 0.0	0.051	−0.5	−1.2 to 0.1	0.108
2	−0.6	−1.0 to 1.0	0.011^b^	−1.0	−1.6 to −0.4	0.002^b^
3	−0.6	−1.0 to 0.1	0.012^b^	−0.6	−1.2 to 0.1	0.082
4	−0.3	−0.8 to 0.1	0.161	−0.4	−1.1 to 0.3	0.304
5	−0.8	−1.2 to −0.5	<0.001^b^	−0.8	−1.4 to −0.3	0.004^b^
6	−0.8	−1.2 to 0.4	<0.001^b^	−0.7	−1.2 to −0.2	0.009^b^
7	−0.9	−1.3 to −0.5	<0.001^b^	−0.9	−1.5 to 0.4	0.002^b^
8	−1.0	−1.4 to −0.6	<0.001^b^	−0.8	−1.4 to 0.3	0.004^b^
9	−0.9	−1.3 to −0.5	<0.001^b^	−0.8	−1.3 to −0.2	0.006^b^
1−9	−0.7	−1.0 to −0.4	<0.001^b^	−0.7	−1.1 to −0.2	0.004^b^

a Results are corrected for age, laterality of reconstruction, history of chemotherapy, follow-up time after mastectomy, and breast volume.

b Statistically significant.

### Correlation between Breast Volume and Skin Sensibility

For both groups, no correlation was found between breast volume and SWM values, with *R* = 0.033 for the IBR group and *R*= −0.176 for the AFT group.

## DISCUSSION

This was the first study to investigate breast sensibility in postmastectomy patients following full breast reconstruction with autologous fat transfer compared with an accepted standard: implant-based reconstruction. Results showed a better overall skin sensibility of the breast in AFT patients. Our study suggests that AFT has a positive effect on skin sensitivity in breast reconstructions and should be further researched in larger scaled studies with null measurements.

Previous studies reporting on skin sensibility after breast reconstruction also used SWM for quantitative measurements. As reference, Beugels et al. described SWM values for overall healthy breasts at 2.71 ± 0.40 (*n* = 27).^13^ Studies using SWM have indicated that patients who underwent a lateral thigh perforator flap or DIEP flap showed better sensory recovery with nerve coaptation, describing overall mean SWM values of innervated lateral thigh perforator flaps at 3.77 ± 0.82 and innervated DIEP flaps at 4.32 ± 0.70.^11,27^ In contrast, decreased sensibility was found in IBR patients.^8,28^ Our results are in line with these studies, showing better sensibility for AFT patients as compared with IBR patients. Moreover, SWM values in our AFT group are comparable to those of innervated autologous breast reconstructions (3.84 ± 0.59). It should be noted that our AFT population is smaller than the innervated autologous reconstruction groups. Nevertheless, standard deviation is similar, and these SWM results suggest that AFT could positively alter skin sensibility, because they show values similar to those of innervated flaps.

A rationale for improved sensibility following AFT to the breast could be the presence of adipose-derived mesenchymal stem cells (ADMSCs).^29,30^ ADMSCs play a key role in the survival of autologous fat grafts, as they exert angiogenic and adipogenic features. These cells are thought to be beneficial in nerve regeneration because they deliver growth factors and prevent neural scar formation.^20,21,29,31,32^ Moreover, research shows an improvement of sensory recovery after nerve injury, when treated with ADMSCs.^31^ The former statements show that AFT has been proven useful as an alternative treatment for localized peripheral neuropathic pain, and this study is the first one showing that AFT could also promote nerve regeneration and, thus, sensibility in postmastectomy patients.^20^

It is described that sensory innervation of the breast skin comes from lateral and anterior cutaneous branches of the intercostal nerves.^33^ When adjusted for age, follow-up time after mastectomy, history of chemotherapy, and breast volume, SWM values did not differ between the groups for the breast regions upper medial, lower lateral, and upper lateral. It is possible that this indifference in sensibility is attributable to the anatomical nerve distribution of the breast. When the mastectomy is performed, these described neural branches are transected. It could be that nerve regeneration starts laterally and anteriorly, correlating to anatomical breast regions lateral and medial; thus, these could be the first and best-recovered regions of the breast. Results for these specific regions are consistent with literature findings showing best sensory recovery scores for upper medial/lateral and lower medial/lateral regions for all investigated breast reconstruction techniques, independent of nerve coaptation.^8,11,13^ Still, large differences (*P* < 0.001) of sensibility of the remaining areas between the reconstruction groups cannot be ignored.

Even so, it is also possible that nerve regeneration in the breast needs more time. As described by Beugels et al., the final result of nerve coaptation is not found earlier than at 24 months postoperatively.^11^ It could be that our study population needed more time to recover and that a final result could be found 2 years after the final surgery.

Concerning lower SWM scores for IBR patients, a reason for diminished sensibility could be mechanically induced because of stretch of the pectoralis muscle and/or skin with the placement of an implant, consequently causing impediment of nerve recovery.^8^ However, in our study, we found no statistical relation between increased volume and SWM values. Another rationale for diminished sensibility in IBR patients could be the minimal fat layer or extensive scar tissue, both hindering nerve growth. A hypothesis for improved sensory recovery in autologous tissue could be that, unlike for implant-based reconstructions, there is a possibility for neural sprouting in transplanted tissue or adipose tissue.

Despite promising results, this study had limitations, including small sample size in both groups and lack of null measures, being SWM values before starting reconstruction (after mastectomy). Because this study does not include baseline sensibility of participants, no statement can be given on improvement or deterioration of sensibility following IBR or AFT following mastectomy. Nevertheless, the results that we found are comparable to the ones reported in the literature, which have clearly reported improvement of sensibility over time after the mastectomy procedure. In addition, the lack of null measurements prevented the possibility of analyzing the effects of AFT on pain. Because we suspect AFT to have a positive effect on pain levels, future studies should include pain measurements (eg, visual analogue scale score ranging from 1 to 10) to evaluate potential reduction of pain levels after AFT. We also suggest future research to include the type of mastectomy performed (if different methods are included) and to include history of lymphadenectomy in their analysis because this could also affect the extent of preexistent neural trauma. In our study, the effect of these variables could not be researched.

It remains questionable whether these positive results suggesting AFT breast reconstruction are attributable to healing and self-renewal benefits of autologous grafts or can simply be explained by restricting aspects of the IBR itself. Even so, AFT is described to have beneficial effects on neuropathic pain. Possible benign effects of AFT on sensibility should be further explored to add to the list of advantages and feasibility of AFT in breast reconstruction.

## DISCLOSURE

The authors have no funding, financial relationships, or conflicts of interest to disclose.

## ACKNOWLEDGMENTS

The BREAST trial was funded by ZonMw: The Netherlands Organization for Health Research and Development (grant no. 80-83700-98-15505). The funder did not have any authority over any of the study-related activities, consisting of data collection, data management, data analysis, interpretation of results, and writing the report or submission for publication. The authors would like to thank all BREAST trial patients who participated in this study; the Breast Cancer Association of The Netherlands; and all investigators, patient advisors, participating hospitals, referring doctors, and institutions involved in this study, such as the Dutch Health Institute and Health Insurance Alliance Netherlands. They would also like to express their gratitude to Ava Krueger, the medical and scientific illustrator of the included figures.

## Supplementary Material


