# Supplementary material for: Superior Sensibility after Full Breast Reconstruction with Autologous Fat Transfer
Source: Plast Reconstr Surg. 2023 Apr 28;153(2):316–23. doi: 10.1097/PRS.0000000000010619 (PMC10802979; doi:10.1097/PRS.0000000000010619)
Supplement: Supplementary file 2 [file prs-153-316-s002.pdf]

Appendix, Supplemental Digital Content 2. Inclusion and exclusion criteria for the BREAST-trial

Inclusion Criteria
- Female gender<br>- Age 18 years or older<br>- Has been a candidate in the history, or is a candidate for a mastectomy in the near future<br>- Patients undergoing a preventive mastectomy<br>- It is the patient's choice to undergo breast reconstruction<br>- Patient wants to participate in this study<br>- Patient is able to wear the BRAVA® device
Exclusion criteria
- Active smoker or history of smoking 4 weeks before surgery<br>- Current drug abuse<br>- History of allergy to lidocaine<br>- History of silicone allergy<br>- 4 weeks or less after chemotherapy<br>- History of radiation therapy in the breast area<br>- Oncological treatment includes radiotherapy after mastectomy<br>- Kidney disease<br>- Steroid-dependent asthma (daily or weekly) or other diseases<br>- Immune-suppressed or immune-compromised disease<br>- Uncontrolled diabetes<br>- BMI> 30<br>- Large breast size (i.e. larger than cup C), unless the patient chooses to reduce the contralateral side towards cup C<br>- Extra-capsular silicone leaking from the encapsulated implant as a result of previous breast reconstruction<br>- The plastic surgeon treating the patient has serious doubts about the patient's compliance.
