# Supplementary figures and images for: Superior Sensibility after Full Breast Reconstruction with Autologous Fat Transfer
Source: Plast Reconstr Surg. 2023 Apr 28;153(2):316–23. doi: 10.1097/PRS.0000000000010619 (PMC10802979; doi:10.1097/PRS.0000000000010619)

### Appendix, Supplemental Digital Content 3. Study flowchart

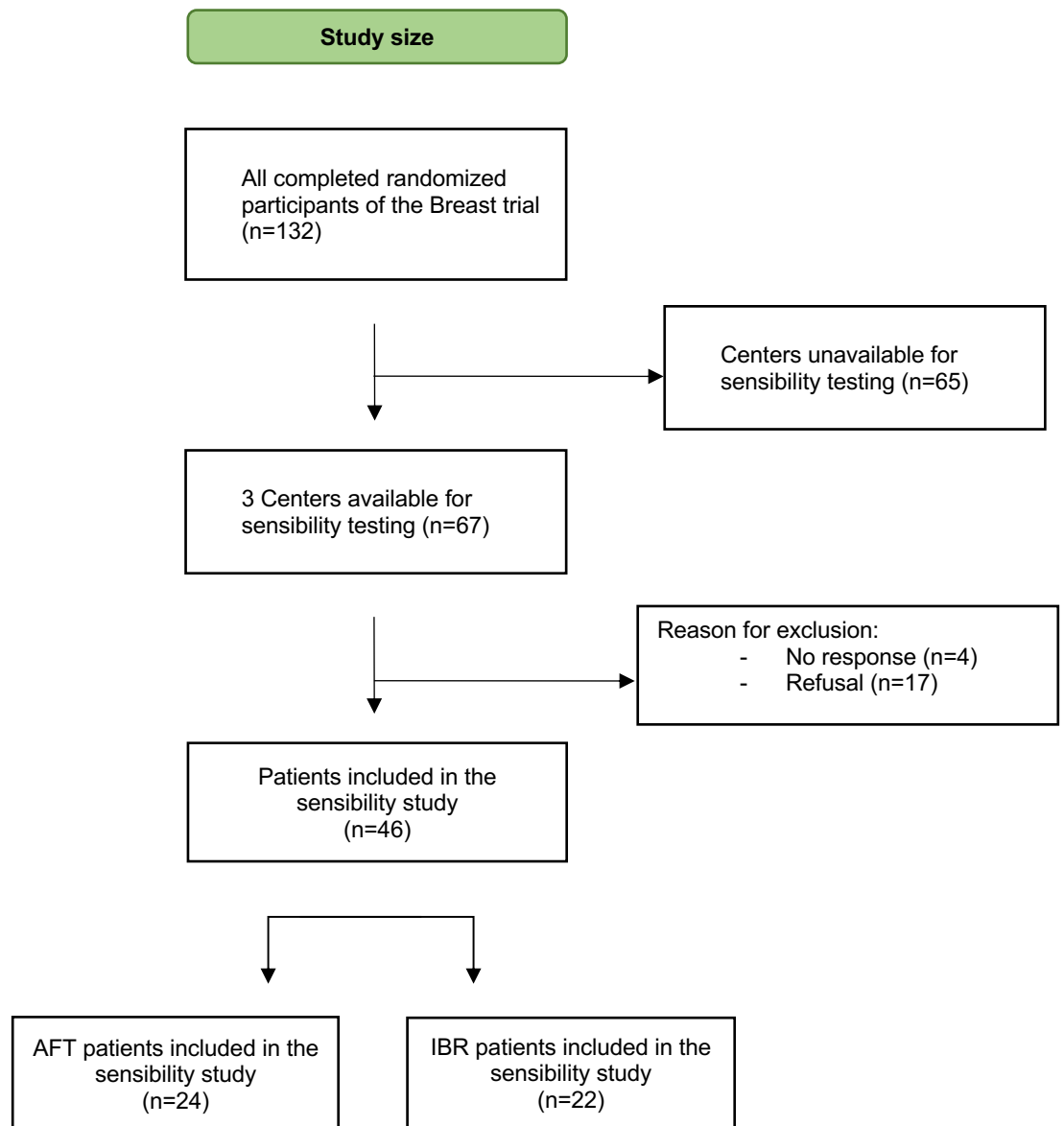

Supplement: Supplementary file 3 [file prs-153-316-s003.pdf]
